# A Density Functional Theory for the Average Electron Energy

**DOI:** 10.1021/acs.jctc.2c00899

**Published:** 2023-01-24

**Authors:** Stefano Racioppi, Phalgun Lolur, Per Hyldgaard, Martin Rahm

**Affiliations:** ^†^Department of Chemistry and Chemical Engineering, ^‡^Department of Microtechnology and Nanoscience—MC2, Chalmers University of Technology, Kemigården 4, Gothenburg, 41296, Sweden

## Abstract

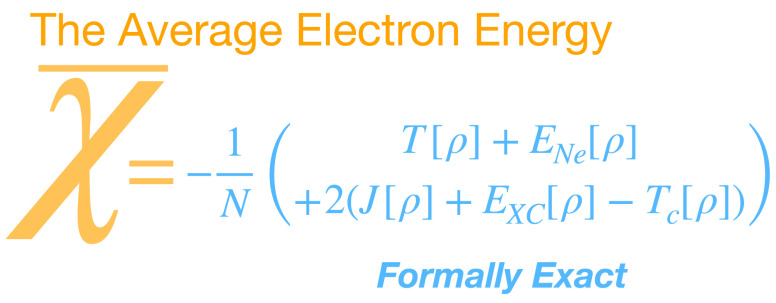

A formally exact density functional theory (DFT) determination of the average electron energy is presented. Our theory, which is based on a different accounting of energy functional terms, partially solves one well-known downside of conventional Kohn–Sham (KS) DFT: that electronic energies have but tenuous connections to physical quantities. Calculated average electron energies are close to experimental ionization potentials (IPs) in one-electron systems, demonstrating a surprisingly small effect of self-interaction and other exchange-correlation errors in established DFT methods. Remarkable agreement with ab initio quantum mechanical calculations of multielectron systems is demonstrated using several flavors of DFT, and we argue for the use of the average electron energy as a design criterion for density functional approximations.

## Introduction

Kohn–Sham (KS) density functional theory (DFT) is the veritable engine of computational chemistry, physics, and materials science. The linchpin idea behind this successful theory is the description of an in principle exact electron density using a set of fictitiously noninteracting electrons, the occupied KS-orbitals. The associated well-known challenges with this approach are several. For example, a self-interaction error arises as a consequence of describing the total energy as a nonexact functional of the electron density,^1^ and corresponds to the unphysical interactions of electrons with themselves. Self-interaction is related to more general delocalization and static correlation errors,^2^ which, together with incorrect asymptotic behaviors of exchange-correlation (XC) potentials,^3^ are important limitations of DFT. A clear physical interpretation of orbital energies is also missing in DFT.^4^ It is only the highest occupied molecular orbital (HOMO) energy that allows for an interpretation via Janak’s theorem,^5^ as a sudden photoionization excitation energy (the first IP).^6^ Resolution of the frontier-orbital energy problem (getting the first IP right) is in practice linked to a correct functional description of the derivative discontinuity and fractional-particle occupations^7^ as well as to the gap renormalization at the molecular-metal interfaces.^8^ Significant advances have been made in these directions through, for example, the development of screened and range-separated hybrid XC-functionals.^9−13^ Impressive agreements with semiconductor band gaps^14−16^ and low-order experimental IPs of molecules^8,17^ are possible in a generalized Kohn–Sham (GKS), range-separated-hybrid^13,16,18−21^ framework.

In this work, we present a DFT determination of the *average* electron energy, χ̅. We then point to the use of χ̅ as a design criteria and quality indicator for density functional approximations. The DFT-based theory we outline is formally exact and does not directly relate to individual photoionization processes nor orbital energies, as is discussed in Appendix A. Our evaluation of χ̅ quantifies the average energy of all electrons in the unperturbed electronic ground state. To explain our KS-orbital-energy-free evaluation of electrons, we first take a step back or away from DFT.

## Theoretical Basis

The average electron energy is an inherent physical property of any system of electrons, for example molecules, and it can be generally defined in *ab initio* quantum chemistry in terms of one-electron energies (*E*_1*e*_) and multielectron energies (*E*_*ee*_),
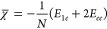
1where *N* is the total number of electrons. In eq 1, *E*_1*e*_ sums the total kinetic energy and the attraction to nuclei of all electrons, while *E*_*ee*_ quantifies all repulsion between electrons in the fully interacting system. In nonrelativistic quantum mechanics, the *E*_1*e*_ and *E*_*ee*_ energies can be interpreted as expectation values of corresponding one- and two-electron operators:
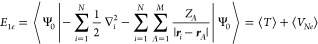
2
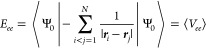
3where Ψ_0_ is the ground state wave function, *i* and *j* denote electrons, *A* denotes nuclei, *M* is the total number of nuclei, *Z*_*A*_ is nuclear charge, and ∇_*i*_^2^ is the Laplace operator. Note in eq 1 that the impact of the electron–electron interactions enters twice in the formal definition of χ̅. This double counting is a natural consequence of the per-electron focus—that χ̅ involves an average over the total number *N* of electrons.

There exist many other ways of expressing χ̅ exactly. For example, in formal many-body physics χ̅ equals the integral of the so-called spectral function weighted by the frequency and the Fermi occupation (see refs (22−25)). In Appendix A we outline an equivalent formulation based on Dyson orbitals,^26−33^ followed by coordinate-based representation in Appendix B.^34,35^

The use of χ̅ in chemistry and physics is broad. Approximations to χ̅ based on orbital energy averaging,^36^ which we will return to discuss, have been productively used to predict properties like Hammett constants,^37,38^ polarizability,^39^ hardness,^40^ electronegativity,^41,42^ electrophilic reactive sites,^43−46^ general chemical reactivity,^47−49^ p*K*_a_,^50^ and solubility.^51^ The average electron energy appears in the theoretical framework of moments of the electron distribution, which is useful for predicting solid state structure (where χ̅ is the first moment).^52^ Interest in χ̅ in chemistry is also rooted in its association with electronegativity,^53−57^ and to its relation with changes in the total energy *E* as
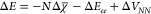
4where *V*_*NN*_ is the nuclear repulsion energy.^54,55^ The energy partitioning of eq 4 is exact within the Born–Oppenheimer approximation and highlights the role of average electron energies in governing chemical and physical processes. The Δχ̅ term has proven helpful for distinguishing and classifying a variety of chemical bond formation reactions,^54,55^ and has even been used to study the effect of compression on the electronegativity of atoms.^58^

### Approximations for the Average Electron Energy,χ̅

Accurate *ab initio* methods such as multireference configuration interaction (MRCI) offers a near-exact evaluation of the average electron energy. However, in practice, such calculations are often prohibitively costly. Most studies of χ̅ are qualitative and rely on some form of approximation separate from the formally exact eq 1. Perhaps the most straightforward computational approximation to eq 1 for molecules is an average over occupied electronic levels, the electronic eigenvalues of a 1-determinant wave function,
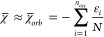
5where *n*_*occ*_ is the number of occupied spin orbitals, and *ε*_*i*_ is the eigenvalue associated with the *i*^th^ spin orbital. Equation 5 is the best possible description of χ̅ within Hartree–Fock (HF) theory, for which the expression summarizes the kinetic and potential energies of electrons alone in the field of the nuclei (*E*_1*e*_ in eq 2) with two times the electron repulsion at this level, i.e., the Coulomb and exchange energies of all electrons (*E*_*ee*_ in eq 3). Within a Hartree–Fock mean field description, Koopmans theorem explicitly connects eq 5 to an average of vertical ionization potentials.^59^ The latter interpretation allows χ̅ to be *estimated* also from experiment, as an average of photoionization energies,
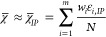
6where *m* is the number of peaks analyzed, *ε*_*i,IP*_ and *w*_*i*_ are, respectively, the vertical ionization energy and the integrated spectral weight associated with the *i*^th^ ionization peak. In the estimates of *χ̅*_*IP*_ that follows we have assumed *w*_*i*_ = 1 and averaged only the ionization peaks of what formally can be viewed as occupied levels in a 1-determinantal picture. The ability to estimate Δχ̅ experimentally, along with Δ*V*_*NN*_ from molecular structures and Δ*E* from thermochemical data, has merited some of us to label eq 4 as an *experimental quantum chemistry* energy partitioning.^55^

We now return to DFT, for which the prevailing approach of evaluating χ̅, by averaging conventional KS-DFT orbital energies (*viz*. eq 5), can be expected to be a poor approximation. KS orbitals are often accurate in reflecting the spatial structure of (and hence matrix elements for) actual excitations and charge transfer.^13,60^ However, the average of the corresponding KS orbital energies is, as we shall outline, not equal to *χ̅.*

### Computing the Exact Average Electron Energy, χ̅, in DFT

Our approach for calculating an in-principle exact and physically motivated χ̅ boils down to a sum of energy terms that does not involve KS orbital energies. We look first at the components of the exact eqs 1–3 so to identify the analogous expressions in KS DFT. The expectation value of the total kinetic energy ⟨*T*⟩ is in KS DFT described as a sum of two terms,
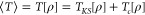
7where *T*_*KS*_[ρ] is the kinetic energy of the noninteracting system (in the case of nondegeneracy this is a single Slater determinant of the KS orbitals) and where the remainder *T*_*c*_[ρ] is the kinetic correlation energy. In other words, *T*_*c*_[ρ] is the difference in kinetic energy between the real interacting electrons we aim to describe and the noninteracting (fictitious) KS orbitals. The division of eq 7 reflects the breakthrough provided by the KS scheme,^61−63^ but is also a consequence of there being no direct way to exactly compute the actual kinetic energy ⟨*T*⟩ in DFT. The correlation component of the kinetic energy is typically handled implicitly, as part of the total XC energy *E*_*xc*_.^61,62^ Undoing the division expressed as eq 7 is key to our proposed DFT calculation of average electron energies.

The second expectation value at the right-hand side of eq 2 describes the energy of the electrons in the field generated by the nuclear charges. Because DFT is formulated in terms of the ground state density, the coupling between the external potential and the density takes a simple (linear) form. This nuclear-electron attraction *E*_*Ne*_[ρ] energy can be evaluated exactly as
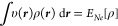
8where υ(***r***) is the potential experienced by electrons interacting with a set of nuclei. The expectation value of the *E*_*ee*_ energy in eq 3 is in DFT terminology expressed as a sum of terms,
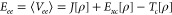
9where *J*[ρ] denotes the Hartree or mean-field approximation to *E*_*ee*_ as calculated in DFT,
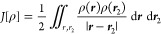
10

We remind that one part of the function of the *E*_*xc*_[ρ] energy is to compensate for the inclusion of electron-self-interaction in *J*[ρ]. In other words, whereas there is a criterion *i* < *j* in eq 3 that is carried over to HF approximations, the Hartree term is corrected differently in DFT.^14−19,64^ The formulation of *E*_*ee*_ in eq 9 follows from the definition of the XC energy, *E*_*xc*_[ρ] = ⟨*T*⟩ + ⟨*V*_*ee*_⟩ – *J*[ρ] – *T*_*KS*_,^62,65^ since *T*_*c*_[ρ] is just the difference between the actual kinetic energy and *T*_*KS*_, the kinetic energy of a single Slater determinant of KS orbitals.^62^

By combining eqs 1–3 with eqs 7–10, we arrive at the formally exact DFT-based determination of the averaged electron energy:
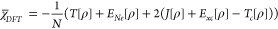
11

Equation 11 is *mostly* given in terms provided by generic molecular and periodic DFT codes. We do, however, need a separate step for computing the *T*_*c*_[ρ] term, which we outline in the SI. Fortunately, the *T*_*c*_[ρ] term is often small compared to the total XC energy in molecules, where exchange dominates. In cases where this assumption holds in practice we may proceed with an approximate characterization,
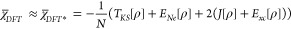
12where all terms are directly available in standard molecular DFT codes. We shall use this approximation for the analysis below because we have documented that, for the set of investigated molecules, the ratio of *T*_*c*_ and *E*_*xc*_ is at most 6% (Table S1).

The *χ̅*_*DFT*_ and *χ̅*_DFT*_ quantities are both notably different from HF and KS orbital-based approximations, χ̅_*orb*_^*HF*^ and χ̅_*orb*_^*KS*^, commonly computed in chemistry from eq 5.^50,52,57,58,66,67^Equation 5 and the exact eqs 1 and 11 are *identical* at the HF level. However, an averaging over KS orbital energies also reflects a density-weighted integral of the XC potential, *υ*_*xc*_(***r***),^61^
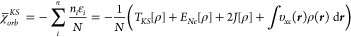
13

Equation 13 differs from both the formally exact eq 11 and the approximate eq 12 by nonzero quantities that arise from the use of the XC potential in DFT:^61,68^
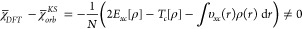
14
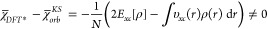
15We note that *υ*_*xc*_(*r*) is given as the functional derivative of the exchange-correlation energy *E*_*xc*_[ρ] that can itself be expressed as a product of the density and the so-called exchange correlation hole *ρ*_*xc*_.^27,28,69−72^ One part of *υ*_*xc*_(*r*), often termed υ_*xc*_^*resp*^(*r*), is set by the functional derivative of *ρ*_*xc*_ and should produce a discontinuity when the electron occupation is changed across integer values.^16−19,26−30,32^ All standard functional approximations more-or-less fail in correctly describing υ_*xc*_^*resp*^(*r*)—i.e., they fail to have a proper account of the derivative discontinuity. Nonetheless, that component of the effective potential helps to set the orbital energies in Kohn–Sham DFT calculations.^61,68^ Effectively, the last term of the right-hand side of eqs 14 or 15 is incorrectly described but nonetheless essential for determining the KS orbital energies and hence also χ̅_*orb*_^*KS*^.

Our evaluation of *χ̅*_*DFT*_ is only weakly sensitive to derivate-discontinuity limitations in the *υ*_*xc*_(*r*) description since we base our evaluation of *χ̅*_*DFT*_ directly on *E*_*xc*_[ρ]. Of course, *χ̅*_*DFT*_ is still sensitive to errors that exist in the electron density ρ and to those that remain in present-day XC functional *E*_*xc*_[ρ]. Use of a hybrid functional, like in refs (9−17), for computations of the density is therefore generally expected to improve the actual *χ̅*_*DFT*_ evaluation.

In brief, we have *χ̅*_*DFT*_ ≈ *χ̅*_DFT*_ ≠ χ̅_*orb*_^*KS*^, three methods through which the average electron energy χ̅ can be evaluated using DFT. They all deliver at varying degrees of approximation for even our formally exact expression relies on nonexact XC energy functionals.

In what follows, we focus on the analysis of *χ̅*_DFT*_ and comparisons with estimates of χ̅ from HF and KS-orbitals (χ̅_*orb*_^*HF*^ and χ̅_*orb*_^*KS*^*viz*. eqs 5 and 13), MRCI calculations (*χ̅*^*MRCI*^*viz*. eqs 1–3), and with experimental photoionization data (*χ̅*_*IP*_*viz*. eq 6). To facilitate for a thorough comparison between *χ̅*_DFT*_ and χ̅_*orb*_^*KS*^, we rely on two well-known XC functionals, PBE^73^ (providing χ̅_DFT*_^PBE^ and χ̅_*orb*_^*PBE*^) and B3LYP^9,10^ (providing χ̅_DFT*_^B3LYP^ and χ̅_*orb*_^*B3LYP*^). We will at times refer to our *χ̅*_DFT*_-values as *XC corrected* average electron energies. We extract *χ̅*_DFT*_ values from conventional output files of standard quantum-chemistry codes (see Supporting Information for computational details). We have made an in-house update of the postprocessing ppacf code^65^ of the Quantum Espresso (QE) suite^68^ for planewave computations of both the kinetic-correlation energy *T*_*c*_ and *χ̅*_*DFT*_ for molecular and extended systems. This ppacf code update is available upon request and will be released to the open-source QE suite.

## Results and Discussion

### One-Electron Systems

The self-interaction error of DFT is most apparent in systems of only one electron. The XC energy functional must here produce an effective local XC potential that exactly cancels what is clearly a spurious Hartree or mean-electron-field energy contribution to *J*[ρ].^1^ For one-electron systems *χ̅ should* equal the total energy of the system.

Table 1 shows χ̅ calculated in different ways for three one-electron systems: H, He^+^, and H_2_^+^. Also shown are experimental references, *χ̅*_*IP*_, which in these cases are nothing more than the single ionization potential of the molecule or ion in question. Note in Table 1 the clearly unphysical energies of the KS-orbitals (χ̅_*orb*_^*PBE*^ and χ̅_*orb*_^*B3LYP*^ are here the negative of the energies of occupied KS energy levels). For example, the KS-orbital of H is attributed an energy of 7.6 and 8.8 eV with PBE and B3LYP, respectively. The actual energy of an electron in H is exactly the ionization potential of the atom, 13.598 eV.

**Table 1:** Average Electron Energies χ̅ of One-Electron Systems in eV·e^–1^, Estimated at Varying Levels of Approximation

	χ̅_*orb*_^*PBE*^	χ̅_*orb*_^*B3LYP*^	χ̅_DFT*_^PBE^	χ̅_DFT*_^B3LYP^	χ̅_*orb*_^*HF*^	*χ̅*^*MRCI*^	*χ̅*_*IP*_^a^
H	7.574	8.767	13.614	13.745	13.604	13.605	13.598
He^+^	42.038	44.510	54.098	54.356	54.418	54.419	54.418
H_2_^+^	23.772	25.004	30.414	30.450	30.017	30.019	30.005

a Computed from eq 6 and experimental data detailed in the Supporting Information.

The improvement provided by eq 12 is drastic: *χ̅*_DFT*_ is already with a conventional generalized-gradient-approximation (GGA) functional, such as PBE, in near perfect agreement with experiment. The value of χ̅_DFT*_^PBE^ for H is, likely somewhat fortuitously, only 16 meV different from experiment. The hybrid XC functional B3LYP is less affected by self-interaction error overall but overestimates the electron energy in H by 0.14 eV.

### Two-Electron Systems

In Table 2, we next compare two-electron systems: He, H_2_, and H^–^. With a second electron correlation energy is introduced, and we here expect the mean-field picture of HF to fail to some degree. In principle, the energy of two explicitly correlated electrons should be better described by DFT.

**Table 2:** Average Electron Energies χ̅ of two-Electron Systems in eV·e^–1^, Estimated at Varying Levels of Approximation

	χ̅_*orb*_^*PBE*^	χ̅_*orb*_^*B3LYP*^	χ̅_DFT*_^PBE^	χ̅_DFT*_^B3LYP^	χ̅_*orb*_^*HF*^	*χ̅*^*MRCI*^	*χ̅*_*IP*_
H^–^	–3.328	–2.432	1.294	1.563	0.398	1.571	0.754
He	15.734	17.986	25.998	26.552	24.976	26.601	24.587
H_2_	10.378	11.827	17.109	17.478	16.176	17.689	15.980

Indeed, whereas the estimates based on averaging KS orbital energies are far off the mark and sometime predict unbound electrons, the corrected χ̅_DFT*_^PBE^ and χ̅_DFT*_^B3LYP^-values are all slightly larger than the negative of the HF orbital energies, χ̅_*orb*_^*HF*^. In other words, our *χ̅*_DFT*_ evaluations correctly describe electrons that are *more bound* compared to the mean field description. Adding the *T*_*c*_ contributions to extract *χ̅*_*DFT*_ increases the values only marginally (Table S1). The improvements provided by the near exact *χ̅*_DFT*_ is further highlighted when comparing to MRCI results. MRCI introduces, at considerable computational costs, correlation energy contributions which are missed in HF theory. Going forward, *χ̅*^*MRCI*^ (and *not χ̅*_*IP*_) represents our reference values for validating eq 12 in multielectron systems. Whereas photoionization experiments can provide highly accurate measurements of the electron energy in one-electron systems (Table 1), the comparison becomes approximate for multielectron systems.

There are several reasons for why *χ̅*_*IP*_ is only approximately equal to χ̅ for systems of more than one electron (Appendix A). Relaxation of the electronic structure, e.g., spatial contraction of orbitals, upon ionization is the main reason why we should expect measures of *χ̅*_*IP*_ to systematically underestimate the actual average electron energy χ̅.^74,75^ Indeed, in all our data for multielectron systems, our *χ̅*_DFT*_ estimates and near-exact *χ̅*^*MRCI*^ reference values of χ̅ are consistently larger, i.e., show *more strongly bound electrons*, compared to estimates from experimental photoionization, *χ̅*_*IP*_ (Table S1). Ionization of single molecules can, of course, be calculated very accurately, especially with MRCI. However, averaging of such accurate ionization energies, *cf*. eq 6, does *not* equal the average electron energy, χ̅. We explain in Appendix A how the use of Dyson orbitals yields an exact expression for χ̅ that resembles eq 6 but one that contains a sum over infinitely many possible transition energies and associated weights. Whereas observable in principle, evaluating all couplings between an initial state and all eigenstates with one electron less in such a framework requires assumptions, and does not lead to an exact determination in practice.

In DFT, the phenomenon of electronic relaxation upon ionization is commonly couched in terms of the XC discontinuity problem, referring to the XC potential being the same before and after addition or removal of an electron, even though it should not.^7,18,19^ We again stress, due to the ease of misinterpretation, that we do not discuss ionization, but a single-state property, χ̅, which is only approximately related to electron binding, and strictly observable (by analysis of a set of ionization peaks) only for one-electron systems (Table 1).

### Larger Molecules

Table 3 shows comparisons of approximations to χ̅ in larger molecules. The real take-away from Table 3 is the notable agreement in absolute values of χ̅_DFT*_^PBE^, χ̅_DFT*_^B3LYP^, and *χ̅*^*MRCI*^. Our XC-corrected average electron energies *χ̅*_DFT*_ are also systematically larger than χ̅_*orb*_^*HF*^. We can attribute the difference in energy to the missing correlation energy in a HF description.

**Table 3:** Average Electron Energies χ̅ of a Selection of Molecules in eV·e^–1^, Estimated at Varying Levels of Approximation

	χ̅_*orb*_^*PBE*^	χ̅_*orb*_^*B3LYP*^	χ̅_DFT*_^PBE^	χ̅_DFT*_^B3LYP^	χ̅_*orb*_^*HF*^	*χ̅*^*MRCI*^
HF	144.294	148.368	165.107	165.458	163.001	165.352
H_2_O	112.859	116.395	130.826	131.165	129.054	131.152
NH_3_	85.841	88.942	101.305	101.666	99.909	101.658
CH_4_	62.749	65.510	76.053	76.475	75.021	76.485
CO	123.005	126.875	142.145	142.587	140.662	142.451
N_2_	120.140	124.040	139.036	139.541	137.882	139.391
CO_2_	129.585	133.613	149.354	149.834	147.992	149.626
C_6_H_6_	85.49	88.78	101.47	101.91	100.46	a

a Not computationally feasible.

Complementary estimates to χ̅ obtained from the averaging of photoionization peaks, i.e., *χ̅*_*IP*_ values, are in part provided as a small test set for the *experimental quantum chemistry* approach of eq 4 in Table S1. Estimates of *χ̅*_*IP*_ are clearly smaller in absolute terms, compared to *χ̅*_DFT*_, χ̅_*orb*_^*HF*^, and *χ̅*^*MRCI*^, as expected from electronic relaxation effects.^76^ We note, however, that a linear regression of *χ̅*^*MRCI*^ and *χ̅*_*IP*_ in our test set of molecules has a coefficient of determination (*r*^2^) of 0.9998, implying that relative measures, Δχ̅, can be productively approached experimentally (Figure S1).

#### Using χ̅ to Guide Density Functional Development

**Figure 1:**
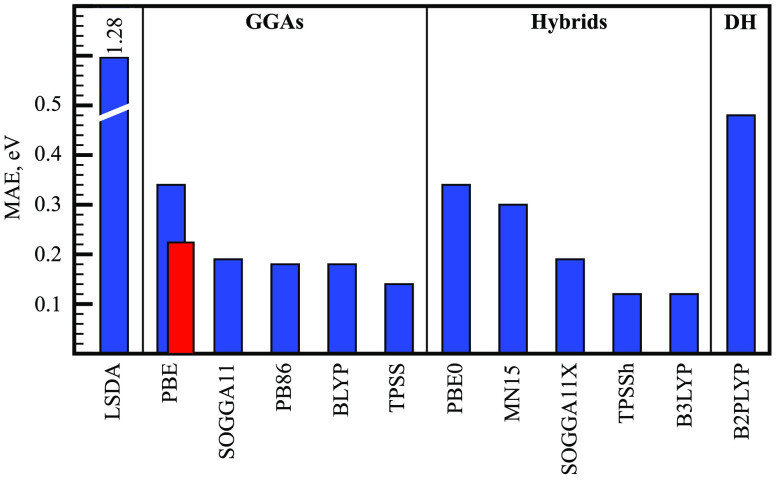
Mean-average error (MAE) of *χ̅*_DFT*_ from *χ̅*^*MRCI*^ computed with a selection of common density functional approximations. DH = double hybrid. The corresponding MAE for formally exact *χ̅*_*DFT*_ is shown for PBE in red.

One way to use more physically motivated average electron energies, such as *χ̅*_DFT*_ (ideally *χ̅*_*DFT*_), is as a metric of quality of density functional approximations. DFT methods are today primarily evaluated against total energies and, to a lesser degree, properties.^77^ The average electron energy χ̅ contains all the terms most challenging in DFT functional design *twice*, those describing electron repulsion and correlation effects. All density functional approximations are associated with errors in both the density and in the computed XC energy for that density. Computations of total energies may therefore appear accurate because of fortuitous cancellations of these kinds of errors. By relying on χ̅ as a quality assessment, we offset such cancellations. Figure 1 shows a selection of common functionals evaluated against the MRCI data of Tables 1–3 (see also Tables S4–S5). Our test set is not exhaustive and relies on the omission of *T*_*c*_ in the definition of *χ̅*_DFT*_. Consequently, Figure 1 data do not necessarily reflect the inherent quality of individual functionals. Instead, Figure 1 is suggestive of an overall high quality of common DFT methods.

Nevertheless, our formal theory now makes it possible to evaluate functional quality not only on DFT total-energy results *E*_*tot*_ but also, independently, on the average-electron energy χ̅. Having two independent assessments provides an option to separate out density-driven functional errors.^13,78^ The comparison is not only possible for molecules, but also, as we show in the SI for general extended systems.

The opportunity for general-system testing presents itself because our theory addresses a fundamental challenge of handling the well-known divergence of Coulomb terms in infinite (ionic) crystals:^79^ The absolute energy position of KS levels cannot be rigorously defined unless we make assumptions for the charge distributions at the far-away bulk-system surfaces. It is a subtle but important point that we here present an exact theory for average electron energy and an evaluation-scheme that is independent of the arbitrarily chosen reference for the KS-orbital energy. Instead, it suffices to know the kinetic energy and the density, which are both set by the shapes of the orbitals (and hence of the electron density), as we detail in the SI. We can therefore complete the formally exact characterizations of χ̅ and analysis of chemical bonding also in general materials.

## Conclusions

The usefulness of conventional KS DFT derives from the construction of correlated electron densities from fictitious noninteracting electronic orbitals. Several of the known problems with this successful theory are, in one way or another, interpretative in nature and related to the tenuous connection between KS orbitals and physical electrons. The purpose of this work is to partially address this issue, by presenting a formally exact KS-orbital-energy-free evaluation of physical electron energies in DFT. Our inspiration for considering the average electron energy derives from chemistry, where more qualitative KS-orbital-energy-based approximations to this quantity have proven useful for analyzing chemical bonding, reactivity, and electronegativity.

A more physical description of electrons is demonstrated in several ways: The average electron energies of H, He^+^, and H_2_^+^ compute as only fractions of an eV away from experimental ionization potentials using conventional GGA functionals. Our approach provides a more bound description of the average electron compared to HF theory in larger molecules, and values consistently resemble those obtained at accurate *ab initio* MRCI level of theory, but at drastically reduced computational costs. This agreement is a testament to how well self-interaction errors are handled in KS DFT. We therefore suggest that the average electron energy can be used as a physically motivated (albeit not easily observable) indicator of quality, and as a constraint, in the design of DFT functionals.

Our theory is straightforwardly implementable together with standard DFT functionals and software and comes at no additional computational cost. Potential utility abounds: More physically motivated electron energies will, for example, provide better predictions of chemical reactivity. Implementation is here exemplified on molecular calculations, but applicability is being developed also for extended systems. A promising avenue to be explored is using average electron energies to constrain KS electronic levels. Such developments may lead to further improvements in the modeling of light-matter interactions, spectroscopy, electron transport, and a range of material properties. Our formal theory is important in the broader context of developing general-purpose XC energy functionals.

## APPENDIX A: A Formally Exact χ̅ Evaluation and Interpretations Based on Dyson Orbitals

We first observe that the Hamiltonian can be expressed as

A1

A2

where *N* denotes the total number of electrons. Equation A1 can be recast by a formal partitioning into a Hamiltonian-component eq A2 that describes electron 1, a term that describes the interactions of electron 1 with all remaining electrons, plus an *N* – 1 Hamiltonian for electrons 2 to *N*:

A3

The set of Dyson orbitals^26−33^ for the fully interacting system is defined as

A4

where Ψ_λ_^*N*-1^ is the set of exact (*N* – 1)-electronic eigenstates, where ***x̅*** = ***r****,****s*** is the (spatial and spin) electron coordinate, with ***s*** = ↑ or ↓. Note that the so-called Dyson orbitals

A5

characterize a coupling from the initial state Ψ_0_^*N*^ to one of infinitely many (*N* – 1)-electronic states that we identify by λ. These orbitals are effectively characterizing single-hole excitations that may have either a spin-↑ and spin-↓ character if we start from a close-shell configuration.

We also observe that the spin-resolved electron density can be expressed as

A6

and that the sum over the infinite in turn integrates to *N*:

A7

Single- and multielectron energies can be expressed as

A8

and

A9

Combining eqs A3, A8, and A9 allows us to recast the χ̅ evaluation as

A10

where we evaluate the first term through Dyson orbitals

A11

In summary we have the formally exact

A12

where *w*_*λ*_ = ∑_σ=↑↓_∫|*d*_*λ*_((*x̅*)|^2^ d***r*** characterizes the weight of the coupling between the initial state and one of infinitely many λ eigenstates of the *N* – 1 system. The energy difference (*E*_λ_^*N*-1^ – *E*_0_^*N*^) in eq A12 can be interpreted as a continuum of excitation energies with corresponding noninteger weights that describe the system with *N* – 1 electrons.

There is, however, no direct relation between the Dyson weights in eq A12 and the finite, discrete set of integrated weights that characterize the experimentally observed ionizations in eq 6. At the single-reference HF and DFT-levels of approximation, the sum in eq A12 does indeed reduce to a finite sum of discrete peaks, as in eq 6, each having an integer weight. This follows because (a) with a single Slater determinant, there is only a limited number of occupied orbitals from which you can extract electrons and (b) in a mean-field description of electron interactions there is no mixing of Slater determinants to reduce the unity weight of each excitation. In a single-reference situation, the Dyson orbitals are also equal to the occupied orbitals.

However, for a fully interacting system, the situation is different. We are then, in principle, working with a superposition of Slater determinants to find the set of possible *N* – 1 eigenstates that enters in eq A12. There is now, in principle, a continuum of possible excitations because each set of *N* – 1 excited states can contain more complex electron configurations set by the superpositions. Consequently, the corresponding weights of individual contributions are reduced. In summary, an evaluation like eq 6 is a natural interpretation of χ̅, but is also an approximation that ignores the fact that the weights in eq A12 are noninteger. We will return in a forthcoming paper to a many-body perturbation theory characterization of χ̅^23^ that in essence treats the right-hand side of eq A12 as an integral over possible single-hole excitation energies. Such an evaluation of χ̅ is also exact and will provide a complementary interpretation of fully interacting systems in which the discrete set of single-reference contributions in eq A12 is both shifted and broadened.

## APPENDIX B: A Coordinate Representation of χ̅

Another way to express and understand χ̅ is in a coordinate representation:

B1

where *τ*_*L*_(***r***) is the kinetic energy density, υ(***r***) is the nuclear potential, ρ(***r***) is the electron density,

B2

is the pair function,^34^ and *ρ*_*XC*_(***r***,***r***_2_) is the exchange-correlation hole density.^81^ The average electron energy χ̅ can be spatially resolved^57^ as

B3

Or, equivalently, it can be expressed in terms of Dyson orbitals,^31^

B4

where *d*_*λ*_(***r***) are Dyson orbitals and *E*_λ_^*N*- 1^ – *E*_0_^*N*^ are corresponding energies as defined in Appendix A. We note that χ̅(***r***), expressed either as eq B3 or B4, depends on the local kinetic energy density *τ*_*L*_(***r***), which is not uniquely defined.^80,81^ This is in contrast to the average electron energy χ̅, for which we are here reporting a formally exact evaluation.

## Supporting Information Available

Additional details on the methodology, theoretical background and supporting data (PDF)
Chart of % errors (XLSX)
