# Supplementary material for: A Density Functional Theory for the Average Electron Energy
Source: J Chem Theory Comput. 2023 Jan 24;19(3):799–807. doi: 10.1021/acs.jctc.2c00899 (PMC9933435; doi:10.1021/acs.jctc.2c00899)
Supplement: Supplementary file 1 — ct2c00899_si_001.pdf [file ct2c00899_si_001.pdf]

Supporting Information

A Density Functional Theory for the Average Electron Energy

Stefano Racioppi[1], Phalgun Lolur[1], Per Hyldgaard[2], Martin Rahm[1]**

[1]Department of Chemistry and Chemical Engineering, [2]Department of Microtechnology and Nanoscience—MC2, Chalmers University of Technology; Kemigården 4, Gothenburg, 41296, Sweden.

Methods

Most PBE,[1,2] B3LYP,[3,4] and Hartree-Fock theory calculations were performed with Gaussian 16, revision B.01,[5] for which energy terms were extracted using the route section "ExtraLinks=L608".[1] We used the Stevens, Basch, Krauss, Jasien and Cundari (SBKJC) Gaussian basis sets and effective core-potentials for all calculations related to KS orbital corrections.[6] Multireference configuration interaction (MRCI) calculations were performed with MOLPRO 2019.2[7] at the internally contracted (IC-MRCI) level,[8] while correlating all valence electrons. Multi-configurational self-consistent field (MCSCF)[9] full valence active space calculations were used to provide the reference orbitals to the IC-MRCI calculations.

All Gaussian and MOLPRO calculations relied on standard correlation-consistent cc-pVQZ basis sets.[10] Evaluations of $\bar{\chi}_{IP}$ were made using photoelectron spectroscopy data from the National Institute of Standard and Technology (NIST) *Chemistry WebBook* and References 11–16, as specified also in Table S1. Ground state geometries were used throughout, as provided by the Computational Chemistry Comparison and Benchmark (CCCBDB) database of NIST.

Evaluations of the $T_c[\rho]$ terms, in $\bar{\chi}_{DFT}$ and discussed below, were done using an update of our PPACF postprocessing code[17] and Quantum Espresso (QE).[18] The modified PPACF code (modification off a general release QE-ppacf 641) is available upon request and a ppacf-update will eventually be released to the open-source QE suite. The PWSCF regular-DFT code of QE was used to determine the electron-structure variation, in Γ-point only calculations with the PBE[1,2] exchange-correlation functional. The QE calculations used a 160 Ry wavefunction energy cut off

[1] Additional information and references on the exchange correlation functionals used can be found at https://gaussian.com/dft/ and https://gaussian.com/overlay3/ .

and relied on optimized norm-conserving Vanderbilt pseudopotentials (ONCV).[19] These calculations thus focus on valence states, omitting 1s electrons for C, N, and O, elements that partake in some of our systems.

Output files for all calculations presented in this work are available through the Swedish National Database (SND): https://doi.org/10.5878/bkth-qw60

Planewave (QE) calculations of $\bar{\chi}_{DFT}$

The planewave code QE allows for the evaluations of $\bar{\chi}_{DFT}$ in neutral systems but not, generally, the orbital-based approximation, *i.e.,* $\bar{\chi}_{orb}$ of Eq. (5) (except in the limit of infinitely large unit cells). Planewave codes such a QE represent the electron density $\rho(r)$ in terms of Fourier components ρ_G with respects to an implicit Bravais lattice defined in a periodic cell. The average electron density is denoted $\bar{\rho}^-$ and QE enforce a vanishing average electrostatic potential by adding $\bar{\rho}^-$ to the ion charge, when computing an ion-potential energy,

$$E'_{eN} = \iint \rho(r) \frac{[\bar{\rho}^- + \sum_i (Z_i e)\delta(r - R_i)]}{|r - r'|} drdr' \tag{S1}$$

and by adding $\bar{\rho}^+ = -\bar{\rho}^-$ to the electron density, when computing a Hartree-energy component,

$$E'_H = \frac{1}{2} \iint \frac{[\bar{\rho}^+ + \rho(r)][\bar{\rho}^+ + \rho(r')]}{|r - r'|} drdr' \tag{S2}$$

As such, the KS orbital energy values are affected by an offset that is dependent on the unit cell-size. All such effects are exactly compensated for in the total energy evaluation.[18]

An apparent but not actual complication for the QE evaluation of $\bar{\chi}_{DFT}$ is that the actual Hartree and electron-Nucleus energies, E_H and E_{eN} diverges in crystals. Fortunately, QE instead works with modified Hartree and electron-nuclear energies, E'_H and E'_{eN}, that remains well behaved and

are directly accessible. In fact, a Fourier-transform representation given by Bravais vectors G and Coulomb matrix elements $4\pi/G^2$ of the set of electrostatic terms permits a complete formal evaluation of the relevant differences,

$$E_{eN} + 2E_H \; - \; (E'_{eN} + 2E'_H) = \bar{\rho}^- \sum_{G\neq 0} \rho_G \frac{4\pi}{G^2} \int e^{iGr} dr = \bar{\rho}^- \sum_{G\neq 0} \rho_G \, \delta_{G,0} = 0, \qquad \text{(S3)}$$

where δ denotes the Kroniker delta. In other words, for calculation of the average electron density there are no adverse effects from relying on the standard redefinition of the potential floor to an arbitrary zero. Whereas this standard planewave-DFT trick impacts evaluations of $\bar{\chi}_{orb}^{KS}$, it does not matter for evaluations of $\bar{\chi}_{DFT}$ and $\bar{\chi}_{DFT*}$.

In effect, we can, for neutral systems, read off the components of Eq. (12) either directly from the QE PWSCF output file, or by using an in-house formulation of the PPACF post-processing code. We can also use PPACF to complete the full $\bar{\chi}_{DFT}$ determination (including the effects of the kinetic correlation term) for a given XC functional approximation. In doing so, we are using the same code and subroutines in both the PWSCF runs (that determines the electron density) and in the PPACF code (post processing). However, as this all-QE approach relies on pseudopotentials, we can then only directly compare results with an MRCI description in those cases where we have retained all electrons (*i.e.,* in the case of the ONCV pseudopotentials, for constituent elements up to Be), and we defer further discussions to forthcoming work.

Nature and impact of the kinetic-correlation energy, $\mathbf{T_c[\rho]}$

We note that the magnitude of the kinetic correlation term is exactly specified by the choice of the XC energy functional. A system-specific but complete determination of $T_c[\rho]$ is possible by a formal scaling argument[17,20] that holds also when making XC energy approximations. One only

needs to track (numerically in PPACF[17]) a coordinate scaling of the spatial variation in the self-consistent electron density solution for a given functional. In short, we have access to an exact computation of the $T_c[\rho]/|E_{xc}[\rho]|$ ratio (discussed below) through a high-quality PWSCF determination of the density variation in our set of molecular systems (Table S1), and can hence determine $\bar{\chi}_{DFT}$.

Table S1 shows that the ratios $|T_c[\rho]/|E_{xc}[\rho]|$ are indeed small for the investigated set of systems. We are in this table comparing terms computed using our QE valence-electron density descriptions (for which all electrons are included only for H and He systems). A full PBE-evaluation of $\bar{\chi}_{DFT}$ for all molecules is here achieved by adjusting the main-text $\bar{\chi}_{DFT*}^{PBE}$ description by the value $T_c[\rho]/N$, where N is the number of electrons in the MRCI calculations. We motivate this approach by noting that the high density of 1s orbitals ensures that their correlation-energy (and kinetic-correlation energy) contributions is dominated by exchange. At the same time, because $T_c[\rho]$ remains a small correction, we can use QE-based evaluations to complete an evaluation of $\bar{\chi}_{DFT}$ and compare with our MRCI results.

Retaining the impact of the kinetic-correction energy often brings the PBE description closer to the MRCI results, and we conclude that PBE is a highly consistent XC functional. PBE yields formally exact $\bar{\chi}_{DFT}$ evaluations that consistently track $\bar{\chi}^{MRCI}$ to within a few kcal/mol, never deviating more than 0.2% for the larger molecules in our test set (table S1). The deviation is up to an order of magnitude larger in H, H_2, H_2^+, He^+, He, and H^-, where we expect significant self-interaction. We cannot rule out that part of this error is due to the use of a pseudopotential description for these 1s systems in QE. We must also expect that some degree of systematic error in our $\bar{\chi}_{DFT}$ assessment arises from combining results obtained in two codes. QE and Gaussian

will, in principle, produce slightly different wavefunctions and hence electron-density descriptions even for the same density functional.

Comparing Approximations of $\bar{\chi}$

The $\bar{\chi}_{DFT}$ and $\bar{\chi}_{DFT*}$ quantities are both intrinsically different from the HF and KS orbital-based approximations $\bar{\chi}_{orb}^{HF}$ and $\bar{\chi}_{orb}^{KS}$ that can be computed from Eq. (5). We have demonstrated that Eq. (5) and the exact Eq. (1) are identical at the Hartree-Fock (HF) level, *i.e.*, quantities that we would call $\bar{\chi}_{orb}^{HF}$ and $\bar{\chi}^{HF}$ are the same because there is no correlation energy in play. The situation is different with DFT. When KS eigenvalues are inserted into Eq. (5), the resulting quantity $\bar{\chi}_{orb}^{KS}$ can be expressed as,

$$\bar{\chi}_{orb}^{KS} = -\frac{1}{N}\sum_{i}^{n} n_i\varepsilon_i = -\frac{1}{N}\left(T_{KS}[\rho] + E_{Ne}[\rho] + 2J[\rho] + \int v_{xc}(\boldsymbol{r})\rho(\boldsymbol{r})d\boldsymbol{r}\right), \quad \text{(S4)}$$

where ε_i and n_i denote the eigenvalue and the occupation number associated to the ith orbital and the term $v_{xc}(r)$ in the last integral is the applied XC potential.[21] Eq. S4 (or 13) can be derived from the definition of the energy in KS DFT, which solves,

$$[-\nabla^2 + v_{Ne}(\boldsymbol{r}) + v_H(\boldsymbol{r}) + v_{xc}(\boldsymbol{r})]\psi_i(\boldsymbol{r}) = \varepsilon_i\psi_i(\boldsymbol{r}), \quad \text{(S5)}$$

where $v_H(\boldsymbol{r}) = \int \frac{\rho(r')}{|\boldsymbol{r}-\boldsymbol{r}'|} d^3\boldsymbol{r}'$.

Multiplication by $\psi_i(\boldsymbol{r})^*$, integration over the volume of a molecule (or unit cell in an extended calculation) and summing over *i* occupied KS spin orbitals gives:

$$T_{KS}[\rho] + E_{Ne}[\rho] + 2J[\rho] + \int v_{xc}(\boldsymbol{r})\rho(\boldsymbol{r})d\boldsymbol{r} = \sum_{i}^{n} n_i\varepsilon_i, \quad \text{(S6)}$$

where

$$J[\rho] = \frac{1}{2}\int v_H(\boldsymbol{r})\rho(\boldsymbol{r})d\boldsymbol{r}. \tag{S7}$$

Equation (S4) differs from both the formally exact Eq. (11) and the approximate Eq. (12) by non-zero quantities that arise from the nature of the XC potential in DFT:[18,21]

$$\overline{\chi}_{DFT} - \overline{\chi}_{orb}^{KS} = -\frac{1}{N}\left(2E_{xc}[\rho] - T_c[\rho] - \int v_{xc}(r)\rho(r)dr\right) \neq 0, \tag{S8}$$

$$\overline{\chi}_{DFT*} - \overline{\chi}_{orb}^{KS} = -\frac{1}{N}\left(2E_{xc}[\rho] - \int v_{xc}(r)\rho(r)dr\right) \neq 0. \tag{S9}$$

The approximate expression Eq. (12) differs from the formally exact Eq. (11) only by the inclusion of $T_c[\rho]$. Because $T_c[\rho]$ is typically relatively small,[17,20,22] $\bar{\chi}_{DFT}$ and $\bar{\chi}_{DFT*}$ are similar quantities, that is,

$$\bar{\chi}_{DFT} \approx \bar{\chi}_{DFT*} \neq \bar{\chi}_{orb}^{KS}. \tag{S10}$$

We note that $v_{xc}(r)$ has a term that is singular and expresses the sensitivity of the electron-electron correlation function to density evaluations. The weight of that singular behavior can be isolated from the expression of Eq. (S8) or (S9) (see Refs. (22–27))

Experimental Estimates of $\bar{\chi}$

The last column of Table S1 shows estimates of $\bar{\chi}$ from averaging of photoionization energies, i.e.,

$$\bar{\chi} \approx \bar{\chi}_{IP} = \sum_{i}^{m} \frac{w_i \varepsilon_{i,IP}}{N}, \tag{S11}$$

where m is the number of peaks analyzed, $\varepsilon_{i,IP}$ and w_i are, respectively, the vertical ionization energy and the spectral weight associated to the ith ionization peak. We stress that these data should *not* be considered as an accurate estimate of $\bar{\chi}$ (for that we rely on $\bar{\chi}^{MRCI}$). As explained in the main text, relaxation of the electronic structure, *e.g.*, spatial contraction of orbitals upon ionization, is the main reason why we should expect measures of $\bar{\chi}_{IP}$ to systematically underestimate the actual average electron energy $\bar{\chi}$. In our estimates of $\bar{\chi}_{IP}$ we have assumed $w_i = 1$ and averaged only the main ionization peaks of what formally can be viewed as occupied levels in a 1-determinatal picture. Whereas the absolute values derived from measurement are offset as expected, there is nevertheless a striking agreement in trends between theory and experiment (Figure S1).

Table S1: Kinetic-correlation energy $T_c[\rho]$, exchange-correlation energy $E_{xc}[\rho]$, and the percentage ratio $|T_c[\rho]/|E_{xc}[\rho]||$ calculated with PBE for a selection of molecules and atoms. Also shown is a comparison between the resulting formally exact $\bar{\chi}_{DFT}^{PBE}$ description and a MRCI description, $\bar{\chi}^{MRCI}$. Averages of photoionization peaks, $\bar{\chi}_{IP}$, is a poorer but experimentally accessible estimate of $\bar{\chi}$.

	$T_c[\rho]$ [eV]	$E_{xc}[\rho]$ [eV]	$T_c[\rho]/\|E_{xc}[\rho]$ [%]	$\bar{\chi}_{DFT}^{PBE}$ [eVe^{-1}]	$\bar{\chi}^{MRCI}$ [eVe^{-1}]	Deviation[b] [%]	$\bar{\chi}_{IP}$[c] [eVe^{-1}]
H	0.143	-8.352	1.71	13.757	13.605	1.1	13.598 [d]
He^+	0.208	-14.987	1.39	54.306	54.418	-0.2	54.418 [d]
H_2^+	0.164	-9.241	1.78	30.578	30.005	2.1	30.005 [d]
He	1.093	-26.596	4.11	26.545	26.601	-0.2	24.587 [d]
H_2	0.946	-18.766	5.04	17.582	17.689	-0.6	15.980 [11]
H^-	0.645	-11.375	5.67	1.597	1.571	1.7	0.754 [d]
HF	6.380	-134.846	4.73	165.745	165.352	0.2	157.156 [13]
H_2O	5.963	-114.952	5.19	131.422	131.152	0.2	124.914 [12]
NH_3	5.585	-98.543	5.67	101.864	101.658	0.2	95.572 [13]
CH_4	5.174	-86.654	5.97	76.570	76.485	0.1	71.299 [13]
CO	7.469	-138.200	5.04	142.679	142.451	0.2	135.047 [14]
N_2	7.468	-133.506	5.59	139.569	139.391	0.1	132.114 [14]
CO_2	12.622	-235.916	5.35	149.919	149.626	0.2	141.400 [15]
C_6H_6	21.712	-342.187	6.35	102.078	*a*	*a*	94.30 [16]

[a] not computationally feasible. [b] Deviation of $\bar{\chi}_{DFT}^{PBE}$ from $\bar{\chi}^{MRCI}$. [c] References below refer to original experimental data. [d] National Institute of Standard and Technology (NIST) *Chemistry WebBook.*

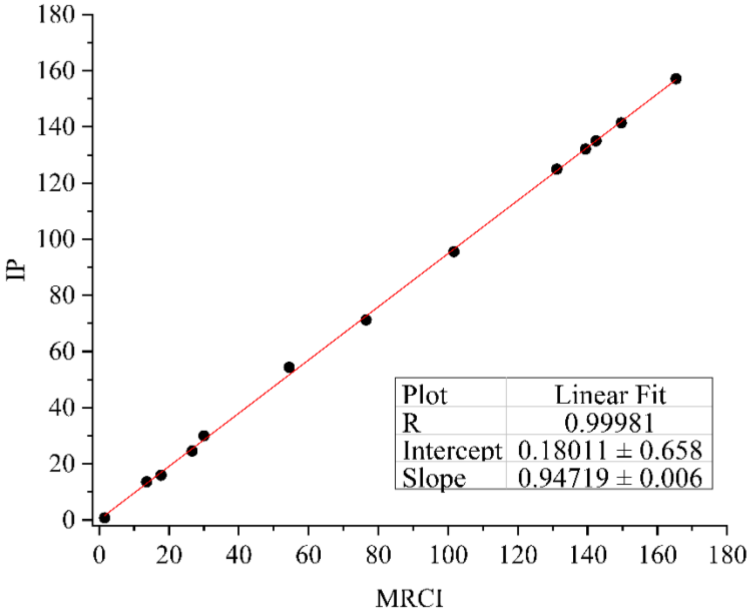

Figure S1. Linear correlation between reference MRCI data, $\bar{\chi}^{MRCI}$, and averages of photoionization peaks, $\bar{\chi}_{IP}$, (IP) values, in [eVe^{-1}].

Table S2: Energy terms in eV for atomic and molecular species. Calculated with Gaussian 16 at the PBE/cc-PVQZ level of theory.

	$E[\rho]$	$T_{KS}[\rho]$	$E_{Ne}[\rho]$	$J[\rho]$	$E_x[\rho]$	$E_c[\rho]$	V_{NN}
H	-13.602	13.495	-27.085	8.370	-8.226	-0.156	0.000
He^+	-54.249	54.095	-108.495	16.838	-16.523	-0.165	0.000
H_2^+	-16.577	17.054	-47.037	9.019	-9.044	-0.190	13.622
H_2	-31.743	31.024	-99.137	35.741	-17.626	-1.168	19.422
H^-	-13.756	15.344	-40.269	24.117	-11.941	-1.007	0.000
He	-78.713	77.759	-183.190	55.206	-27.370	-1.119	0.000
HF	-2731.927	2725.179	-6820.676	1514.207	-282.855	-9.141	141.358
H_2O	-2078.489	2072.433	-5421.273	1272.070	-242.917	-8.862	250.060
NH_3	-1537.811	1531.908	-4245.049	1066.508	-207.895	-8.568	325.285
CH_4	-1101.151	1095.394	-3270.353	893.228	-177.847	-8.166	366.593
CO	-3081.377	3071.345	-8469.362	2078.592	-362.386	-12.209	612.642
N_2	-2978.440	2968.454	-8264.398	2044.387	-357.367	-12.299	642.783
CO_2	-5128.962	5113.010	-15257.267	4037.987	-588.621	-20.127	1586.056
C_6H_6	-6313.907	6282.931	-25709.064	8520.568	-901.098	-37.321	5530.078

Table S3: Energy terms in eV for atomic and molecular species. Calculated with Gaussian 16 at the B3LYP/cc-PVQZ level of theory.

	$E[\rho]$	$T_{KS}[\rho]$	$E_{Ne}[\rho]$	$J[\rho]$	$E_x[\rho]$	$E_c[\rho]$	V_{NN}
H	-13.670	13.549	-27.144	8.402	-8.272	-0.206	0.000
He^+	-54.382	54.203	-108.611	16.881	-16.602	-0.254	0.000
H_2^+	-16.600	16.961	-46.953	9.016	-9.031	-0.214	13.622
H_2	-32.124	31.103	-99.240	35.785	-17.678	-1.516	19.422
H^-	-14.082	15.588	-40.626	24.343	-12.066	-1.321	0.000
He	-79.321	78.099	-183.635	55.400	-27.445	-1.739	0.000
HF	-2734.630	2726.223	-6823.615	1516.355	-282.297	-12.655	141.358
H_2O	-2080.845	2073.153	-5423.317	1273.576	-242.365	-11.952	250.060
NH_3	-1539.931	1532.500	-4246.269	1067.266	-207.464	-11.248	325.285
CH_4	-1103.191	1096.015	-3270.836	893.234	-177.650	-10.547	366.593
CO	-3084.867	3071.849	-8470.645	2079.534	-361.221	-17.027	612.642
N_2	-2981.738	2968.381	-8263.853	2043.900	-355.968	-16.981	642.783
CO_2	-5134.153	5113.253	-15257.328	4038.049	-586.611	-27.572	1586.056
C_6H_6	-6322.563	6285.276	-25710.208	8519.801	-898.821	-48.688	5530.078

Table S4: $\bar{\chi}_{DFT*}$, $\bar{\chi}_{orb}^{KS}$ and relative error compared to $\bar{\chi}^{MRCI}$ of a selection of atoms, ions and molecules in $eV\cdot e^{-1}$, estimated with various density functionals.

See the file Table_S4.xlsx.

Table S5: $\bar{\chi}_{DFT*}$ and $\bar{\chi}_{orb}^{KS}$ computed for the systems reported in Tables 1-3 using a series of different DFT exchange-correlation functionals. The mean absolute error (MAE), mean error (ME), standard deviation (STDV) and root mean square deviation (RMSD) are shown relative to $\bar{\chi}^{MRCI}$.

		$\bar{\chi}_{DFT*}$			$\bar{\chi}_{orb}^{KS}$		
Functional	Type	MAE	ME	RMSD	MAE	ME	RMSD
B3LYP [3,4]	hybrid	0.12	0.07	0.16	10.82	-10.82	11.75
TPSSh [28]	hybrid	0.12	0.01	0.18	11.58	-11.58	12.57
X3LYP [29]	hybrid	0.14	-0.08	0.18	10.65	-10.65	11.55
TPSS [30]	GGA	0.14	0.04	0.19	12.79	-12.79	13.88
O3LYP [31]	hybrid	0.16	-0.09	0.20	11.99	-11.99	13.00
B1LYP [32]	hybrid	0.17	-0.12	0.21	10.29	-10.29	11.15
BLYP [4,33]	GGA	0.18	-0.07	0.21	13.49	-13.49	14.62
BP86 [33,34]	GGA	0.18	0.03	0.22	13.35	-13.35	14.50
LG1LYP [4,35]	hybrid	0.19	0.00	0.22	10.28	-10.28	11.14
SOGGA11 [36]	GGA	0.19	-0.04	0.22	13.27	-13.27	14.43
SOGGA11X [37]	hybrid	0.19	0.09	0.23	8.22	-8.22	8.94
MN15 [38]	hybrid	0.30	-0.25	0.34	10.82	-10.82	12.08
PW6B95 [39]	hybrid	0.31	0.21	0.35	9.86	-9.86	10.68
PBE0 [40]	hybrid	0.34	-0.27	0.37	10.28	-10.28	11.18
PBE [1,2]	GGA	0.34	-0.28	0.37	13.49	-13.49	14.65
M11L [41]	GGA	0.34	0.06	0.42	10.67	-10.67	11.27
B2PLYP [42]	DH[a]	0.48	-0.46	0.53	6.88	-6.88	7.45
LSDA [43,44]	LSDA	1.28	-1.23	1.50	13.55	-13.55	14.81

[a] DH = double hybrid.

References

(1) Perdew, J. P.; Burke, K.; Ernzerhof, M. Generalized Gradient Approximation Made Simple. *Phys. Rev. Lett.* **1996**, *77* (18), 3865–3868.

(2) Perdew, J. P.; Ernzerhof, M.; Burke, K. [ERRATA] Generalized Gradient Approximation Made Simple. *Phys. Rev. Lett.* **1996**, *77* (18), 3865–3868.

(3) Becke, A. D. Density-Functional Thermochemistry.III. The Role of Exact Exchange. *J. Chem. Phys.* **1993**, *98* (7), 5648–5652.

(4) Lee, C.; Yang, W.; Parr, R. G. Development of the Colle-Salvetti Correlation-Energy Formula into a Functional of the Electron Density. *Phys. Rev. B* **1988**, *37* (2), 785–789.

(5) Gaussian 16, R. B. 0.; Frisch, M. J.; Trucks, G. W.; Schlegel, H. B.; Scuseria, G. E.; Robb, M. A.; Cheeseman, J. R.; Scalmani, G.; Barone, V.; Petersson, G. A.; Nakatsuji, H.; Li, X.; Caricato, M.; Marenich, A. V.; Bloino, J.; Janesko, B. G.; Gomperts, R.; Mennucci, B.; Hratchian, H. P.; Ortiz, J. V.; Izmaylov, A. F.; Sonnenberg, J. L.; Williams-Young, D.; Ding, F.; Lipparini, F.; Egidi, F.; Goings, J.; Peng, B.; Petrone, A.; Henderson, T.; Ranasinghe, D.; Zakrzewski, V. G.; Gao, J.; Rega, N.; Zheng, G.; Liang, W.; Hada, M.; Ehara, M.; Toyota, K.; Fukuda, R.; Hasegawa, J.; Ishida, M.; Nakajima, T.; Honda, Y.; Kitao, O.; Nakai, H.; Vreven, T.; Throssell, K.; Jr., J. A. M.; Peralta, J. E.; Ogliaro, F.; Bearpark, M. J.; Heyd, J. J.; Brothers, E. N.; Kudin, K. N.; Staroverov, V. N.; Keith, T. A.; Kobayashi, R.; Normand, J.; Raghavachari, K.; Rendell, A. P.; Burant, J. C.; Iyengar, S. S.; Tomasi, J.; Cossi, M.; Millam, J. M.; Klene, M.; Adamo, C.; Cammi, R.; Ochterski, J. W.;

Martin, R. L.; Morokuma, K.; Farkas, O.; Foresman, J. B.; Fox, D. J. Gaussian16. 2016, p Gaussian, Inc., Wallingford CT.

(6) Stevens, W. J.; Basch, H.; Krauss, M. Compact Effective Potentials and Efficient Shared-Exponent Basis Sets for the First- and Second-Row Atoms. *J. Chem. Phys.* **1984**, *81* (12), 6026–6033.

(7) Werner, H. J.; Knowles, P. J.; Knizia, G.; Manby, F. R.; Schütz, M. Molpro: A General-Purpose Quantum Chemistry Program Package. *Wiley Interdiscip. Rev. Comput. Mol. Sci.* **2012**, *2* (2), 242–253.

(8) Szalay, P. G.; Müller, T.; Gidofalvi, G.; Lischka, H.; Shepard, R. Multiconfiguration Self-Consistent Field and Multireference Configuration Interaction Methods and Applications. *Chem. Rev.* **2012**, *112* (1), 108–181.

(9) Werner, H. J.; Knowles, P. J. A Second Order Multiconfiguration SCF Procedure with Optimum Convergence. *J. Chem. Phys.* **1985**, *82* (11), 5053–5063.

(10) Woon, D. E.; Dunning, T. H. Gaussian Basis Sets for Use in Correlated Molecular Calculations. V. Core-Valence Basis Sets for Boron through Neon. *J. Chem. Phys.* **1995**, *103* (11), 4572–4585.

(11) Kimura, K.; Katsumata, S.; Achiba, Y.; Yamazaki, T.; Iwata, S. *Handbook of He(I) Photoelectron Spectra of Fundamental Organic Molecules*; Japan Scientific Societies Press: Tokio, 1982.

(12) Potts, A. W.; Price, W. C. Photoelectron Spectra and Valence Shell Orbital Structures of Groups V and VI Hydrides. *Proc. R. Soc. A Math. Phys. Eng. Sci.* **1972**, *326* (1565), 181–

197.

(13) Banna, M. S.; Shirley, D. A. Molecular Photoelectron Spectroscopy at 123.3 EV. The Second-Row Hydrides. *J. Phys. Chem.* **1975**, *63* (11), 4759–4766.

(14) Allison, D. A.; Cavell, R. G. Photoelectron Spectroscopy with Zr Mζ(151 EV) Radiation. A Study of the Variation of Relative Photoionization Cross Sections of Molecules Containing First Row Atoms (C, N, O) with Exciting Radiation from HeIto Mg Kαlimits. *J. Chem. Phys.* **1978**, *68* (2), 593–601.

(15) Allan, C. J.; Siegbahn, K.; Gelius, U.; Allison, D. A.; Johansson, G.; Siegbahn, H. ESCA Studies of CO2, CS2 and COS. *J. Electron Spectros. Relat. Phenomena* **1972**, *1*, 131–151.

(16) Carlson, T. A.; Gerard, P.; Krause, M. O.; Grimm, F. A.; Pullen, B. P. Photoelectron Dynamics of the Valence Shells of Benzene as a Function of Photon Energy. *J. Chem. Phys.* **1987**, *86* (12), 6918–6926.

(17) Jiao, Y.; Schröder, E.; Hyldgaard, P. Signatures of van Der Waals Binding: A Coupling-Constant Scaling Analysis. *Phys. Rev. B* **2018**, *97* (8), 085115.

(18) Giannozzi, P.; Baroni, S.; Bonini, N.; Calandra, M.; Car, R.; Cavazzoni, C.; Ceresoli, D.; Chiarotti, G. L.; Cococcioni, M.; Dabo, I.; Dal Corso, A.; De Gironcoli, S.; Fabris, S.; Fratesi, G.; Gebauer, R.; Gerstmann, U.; Gougoussis, C.; Kokalj, A.; Lazzeri, M.; Martin-Samos, L.; Marzari, N.; Mauri, F.; Mazzarello, R.; Paolini, S.; Pasquarello, A.; Paulatto, L.; Sbraccia, C.; Scandolo, S.; Sclauzero, G.; Seitsonen, A. P.; Smogunov, A.; Umari, P.; Wentzcovitch, R. M. QUANTUM ESPRESSO: A Modular and Open-Source Software Project for Quantum Simulations of Materials. *J. Phys. Condens. Matter* **2009**, *21* (39).

(19) Hamann, D. R. Optimized Norm-Conserving Vanderbilt Pseudopotentials. *Phys. Rev. B - Condens. Matter Mater. Phys.* **2013**, *88* (8), 085117.

(20) Levy, M.; Perdew, J. P. Hellman-Feynman, Virial, and Scaling Properties for the Exact Universal Denisty Function. Shape of the Correlation Potential and Diamagnetic Susceptibility for Atoms. *Phys. Rev. A* **1985**, *32* (4), 2010–2021.

(21) Kohn, W.; Sham, L. J. Self-Consistent Equations Including Exchange and Correlation Effects. *Phys. Rev. Rev.* **1965**, *140* (4A), A1133–A1138.

(22) Gritsenko, O. V.; Van Leeuwen, R.; Baerends, E. J. Molecular Exchange-Correlation Kohn-Sham Potential and Energy Density from Ab Initio First- and Second-Order Density Matrices: Examples for XH (X=Li, B, F). *J. Chem. Phys.* **1996**, *104* (21), 8535–8545.

(23) Buijse, M. A.; Baerends, E. J.; Snijders, J. G. Analysis of Correlation in Terms of Exact Local Potentials: Applications to Two-Electron Systems. *Phys. Rev. A* **1989**, *40* (8), 4190–4202.

(24) Gritsenko, O. V.; Baerends, E. J. Effect of Molecular Dissociation on the Exchange-Correlation Kohn-Sham Potential. *Phys. Rev. A - At. Mol. Opt. Phys.* **1996**, *54* (3), 1957–1972.

(25) Baerends, E. J.; Gritsenko, O. V. A Quantum Chemical View of Density Functional Theory. *J. Phys. Chem. A* **1997**, *101* (30), 5383–5403.

(26) Levy, M.; Zahariev, F. Ground-State Energy as a Simple Sum of Orbital Energies in Kohn-Sham Theory: A Shift in Perspective Through a Shift in Potential. *Phys. Rev. Lett.* **2014**, *113* (11), 113002.

(27) Vuckovic, S.; Levy, M.; Gori-Giorgi, P. Augmented Potential, Energy Densities, and Virial Relations in the Weak-A Nd Strong-Interaction Limits of DFT. *J. Chem. Phys.* **2017**, *147*, 214107.

(28) Staroverov, V. N.; Scuseria, G. E.; Tao, J.; Perdew, J. P. Comparative Assessment of a New Nonempirical Density Functional: Molecules and Hydrogen-Bonded Complexes. *J. Chem. Phys.* **2003**, *119* (23), 12129–12137.

(29) Xu, X.; Goddard, W. A. The X3LYP Extended Density Functional for Accurate Descriptions of Nonbond Interactions, Spin States, and Thermochemical Properties. *Proc. Natl. Acad. Sci. U. S. A.* **2004**, *101* (9), 2673–2677.

(30) Tao, J.; Perdew, J. P.; Staroverov, V. N.; Scuseria, G. E. Climbing the Density Functional Ladder: Nonempirical Meta–Generalized Gradient Approximation Designed for Molecules and Solids. *Phys. Rev. Lett.* **2003**, *91* (14), 146401.

(31) Cohen, A. J.; Handy, N. C. Dynamic Correlation. *Mol. Phys.* **2001**, *99* (7), 607–615.

(32) Adamo, C.; Barone, V. Toward Reliable Adiabatic Connection Models Free from Adjustable Parameters. *Chem. Phys. Lett.* **1997**, *274* (1–3), 242–250.

(33) Becke, A. D. Density-Functional Exchange-Energy Approximation with Correct Asymptotic Behavior. *Phys. Rev. A* **1988**, *38* (6), 3098–3100.

(34) Perdew, J. P. Density-Functional Approximation for the Correlation Energy of the Inhomogeneous Electron Gas. *Phys. Rev. B* **1986**, *33* (12), 8822–8824.

(35) Lacks, D. J.; Gordon, R. G. Pair Interactions of Rare-Gas Atoms as a Test of Exchange-Energy-Density Functionals in Regions of Large Density Gradients. *Phys. Rev. A* **1993**, *47*

(6), 4681–4690.

(36) Peverati, R.; Zhao, Y.; Truhlar, D. G. Generalized Gradient Approximation That Recovers the Second-Order Density-Gradient Expansion with Optimized across-the-Board Performance. *J. Phys. Chem. Lett.* **2011**, *2* (16), 1991–1997.

(37) Peverati, R.; Truhlar, D. G. Communication: A Global Hybrid Generalized Gradient Approximation to the Exchange-Correlation Functional That Satisfies the Second-Order Density-Gradient Constraint and Has Broad Applicability in Chemistry. *J. Chem. Phys.* **2011**, *135* (19), 191102.

(38) Yu, H. S.; He, X.; Li, S. L.; Truhlar, D. G. MN15: A Kohn–Sham Global-Hybrid Exchange–Correlation Density Functional with Broad Accuracy for Multi-Reference and Single-Reference Systems and Noncovalent Interactions. *Chem. Sci.* **2016**, *7* (8), 5032–5051.

(39) Zhao, Y.; Truhlar, D. G. Design of Density Functionals That Are Broadly Accurate for Thermochemistry, Thermochemical Kinetics, and Nonbonded Interactions. *J. Phys. Chem. A* **2005**, *109* (25), 5656–5667.

(40) Adamo, C.; Barone, V. Toward Reliable Density Functional Methods without Adjustable Parameters: The PBE0 Model. *J. Chem. Phys.* **1999**, *110* (13), 6158–6170.

(41) Peverati, R.; Truhlar, D. G. M11-L: A Local Density Functional That Provides Improved Accuracy for Electronic Structure Calculations in Chemistry and Physics. *J. Phys. Chem. Lett.* **2012**, *3* (1), 117–124.

(42) Grimme, S. Semiempirical Hybrid Density Functional with Perturbative Second-Order Correlation. *J. Chem. Phys.* **2006**, *124* (3), 034108.

(43) Slater, J. C. *Quantum Theory of Molecular and Solids. The Self-Consistent Field for Molecular and Solids*; McGraw-Hill: New York, 1974; Vol. 4.

(44) Vosko, S. H.; Wilk, L.; Nusair, M. Accurate Spin-Dependent Electron Liquid Correlation Energies for Local Spin Density Calculations: A Critical Analysis. *Can. J. Phys.* **1980**, *58* (8), 1200–1211.
